# Nitrosative stress in Parkinson’s disease

**DOI:** 10.1038/s41531-022-00370-3

**Published:** 2022-08-11

**Authors:** Morgan G. Stykel, Scott D. Ryan

**Affiliations:** 1 The Department of Molecular and Cellular Biology, The University of Guelph, Guelph, ON N1G 2W1 ON Canada; 2 Neurodegenerative Disease Center, Scintillon Institute, 6868 Nancy Ridge Drive, San Diego, CA 92121 USA

**Keywords:** Parkinson's disease, Cellular neuroscience

## Abstract

Parkinson’s Disease (PD) is a neurodegenerative disorder characterized, in part, by the loss of dopaminergic neurons within the nigral-striatal pathway. Multiple lines of evidence support a role for reactive nitrogen species (RNS) in degeneration of this pathway, specifically nitric oxide (NO). This review will focus on how RNS leads to loss of dopaminergic neurons in PD and whether RNS accumulation represents a central signal in the degenerative cascade. Herein, we provide an overview of how RNS accumulates in PD by considering the various cellular sources of RNS including nNOS, iNOS, nitrate, and nitrite reduction and describe evidence that these sources are upregulating RNS in PD. We document that over 1/3 of the proteins that deposit in Lewy Bodies, are post-translationally modified (S-nitrosylated) by RNS and provide a broad description of how this elicits deleterious effects in neurons. In doing so, we identify specific proteins that are modified by RNS in neurons which are implicated in PD pathogenesis, with an emphasis on exacerbation of synucleinopathy. How nitration of alpha-synuclein (aSyn) leads to aSyn misfolding and toxicity in PD models is outlined. Furthermore, we delineate how RNS modulates known PD-related phenotypes including axo-dendritic-, mitochondrial-, and dopamine-dysfunctions. Finally, we discuss successful outcomes of therapeutics that target S-nitrosylation of proteins in Parkinson’s Disease related clinical trials. In conclusion, we argue that targeting RNS may be of therapeutic benefit for people in early clinical stages of PD.

## Introduction

Parkinson’s disease (PD) is the most common movement disorder affecting over 10 million individuals worldwide^1^. PD is characterized by the degeneration of brain cell populations, most notably the dopaminergic neurons emanating from the substantia nigra. Nigrostriatal degeneration correlates with a decline in motor control generally resulting in bradykinesia, rigidity, or tremors. In addition, many non-motor symptoms such as constipation, fatigue or dementia may be concomitant. The neuronal loss in PD is preceded by many phenotypes discussed herein, with a focus on axo-dendritic defects, mitochondrial dysfunction, and synucleinopathy. In this review, we describe how these phenotypes can be attributed to increases in reactive nitrogen species (RNS). We first discuss how RNS is upregulated in PD, we then discuss the effects of RNS on dopaminergic neurons, and then we describe how unchecked RNS leads to aSyn misfolding and Lewy body deposition. In conclusion, we provide an argument that reducing nitrosative stress early in disease may represent a means of delaying phenotypic progression in PD and protecting cells from degeneration.

## Sources of reactive nitrogen species in PD

**Fig. 1:**
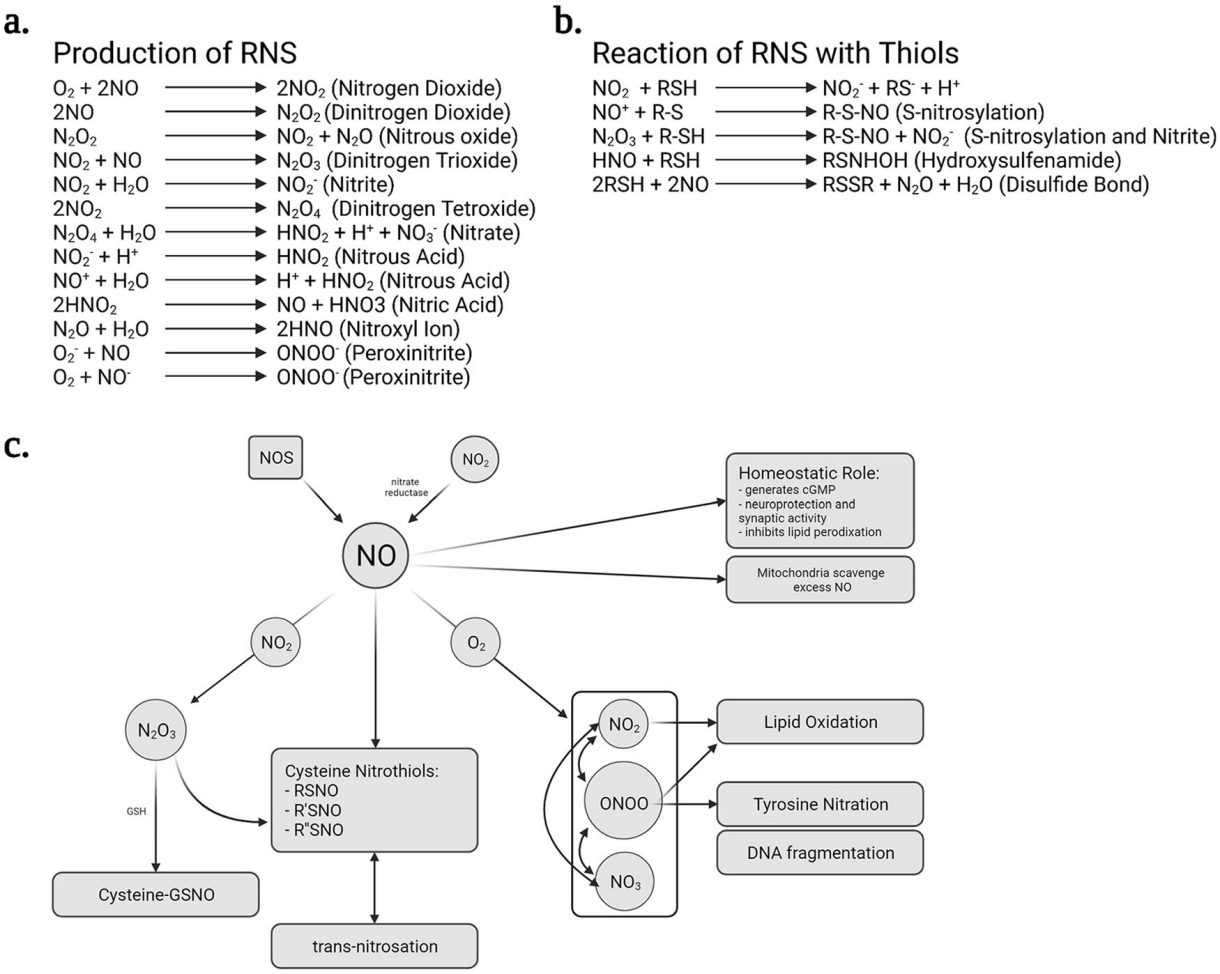
Sources of reactive nitrogen species. Various formulas showing the production of reactive nitrogen species are displayed (**a**). The known reactions between RNS and thiols are also shown (**b**). A cartoon depicting the primary effects of various RNS and their intermediates (**c**). This figure was created using Biorender.

Nitrosative stress results primarily from the over-production of nitrogen based free radicals: nitric oxide (NO^−^) and nitrogen dioxide (NO_2_^−^). These atoms possess unbalanced valence electrons and are therefore highly reactive and prone to filling their outer valance shell with other atoms or molecules. This can lead to production of secondary free radicals such as peroxynitrite (ONOO^−^) and hydroxide anion (OH^−^), as well as toxic non-radicals such as hydrogen peroxide (H_2_O_2_), dinitrogen dioxide (N_2_O_2_), and nitrous acid (HNO_2_) (Fig. 1). Although many of these are present in healthy neurons, several events can lead to their overproduction which, ultimately damages cellular components leading to neuronal dysfunction and increased severity and area of affliction in a time dependent manner^2–4^. It has been reported that PD patients have elevated RNS as indicated from increased levels of nitrite/nitrate in cerebral spinal fluid^5^ and blood^6^. More specifically, in PD it has been reported that white blood cell-neutrophils have higher expression of nNOS and an increased ability to produce excess NO^7^. In fact, Kouti et al. reported that serum levels of nitric oxide positively correlated with increased UPDRS scores (Universal Parkinson’s disease rating scale) and duration of disease regardless of sex or age^6^; however, these findings are contentious^8,9^.

### Increased enzymatic activity of NOS is elevated in the PD brain

**Fig. 2:**
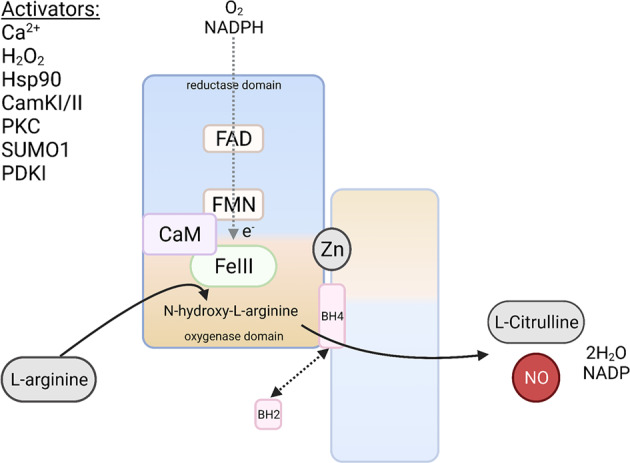
Primary production of NO occurs at the nitric oxide synthase (NOS). NOS may be activated by various ions, proteins, or enzymes. To produce nitric oxide, NOS requires oxygen, NADPH and L-arginine. The reductase (containing FMN) and oxygenase (heme-containing) domain are bound by calmodulin (CaM). To produce nitric oxide, oxygen and NADPH enter the reductase domain facilitating the transfer of the electron from the FAD, to the FMN, to the heme. This electron, along with oxygen, is needed to convert L-arginine into N-hydroxyl-L-arginine (monooxygenase reaction I). Next, a second monooxygenase reaction involving an electron, oxygen molecule and co-factor BH4 occurs to convert N-hydroxyl-L-arginine to L-citrulline and nitric oxide (NO). BH4 is oxidized to BH2, which can then be reduced back to BH4, balancing NOS dimers in an on (coupled)/off (uncoupled) state. NOS subunits are held together by zinc (Zn) ions that bind the oxygenase domains. Output from NOS includes L-citrulline, water, NADP and nitric oxide. This figure was created using Biorender.

Neuronal nitric oxide synthetase (nNOS) is the primary source of nitric oxide (NO) production in neurons. nNOS is a 161 kda enzyme encoded by the NOS1 gene. Each nNOS has an oxygenase and reductase domain connected by a linker that is responsible for calmodulin binding. The generation of NO occurs in two-steps, both requiring oxygen, NADPH, and an nNOS dimer (zinc facilitates the dimerization of nNOS, enabling nNOS activity). First, intracellular Ca^2+^ catalyzes the binding of nNOS to calmodulin and, in the presence of heme, hydroxylates L-arginine to N-hydroxy-L-arginine. In this step, co-factors FAD and FMN aid the electron transfer from the NADPH by the nNOS to the heme. Second, N-hydroxy-L-arginine is oxidized to L-citrulline and NO with the aid of L-arginine and co-factor BH_4_ (Fig. 2). There are at least four splice variants of nNOS: nNOSα, nNOSβ, nNOSγ, and nNOSμ. nNOSα is the most dominant variant, primarily found in neurons. nNOS contains a PDZ domain which allows its interaction with other PDZ-domain containing proteins, thus influencing the cellular localization of NOS. As such, nNOS is often localized at the synaptic membrane due to its PDZ-interaction with PSD95 and PFK-M, for example.

Post-mortem analysis of midbrain samples revealed that nNOS expression is increased in brain regions of PD patients relative to controls^10^. nNOS activity is also upregulated in animal models of PD^11,12^. Similarly, in weaver mice where the spontaneous depletion of dopaminergic neurons occurs, nNOS is increased in the substantia nigra but not in other brain regions^13^. Together these findings implicate nNOS activity in PD pathogenesis. nNOS activity is highly regulated by post-translational modifications and interacting proteins. For example, the phosphorylation of Ser847 is inhibitory, while de-phosphorylation of Ser847 stimulates nNOS activity^14^. Perhaps it is not surprising that docosahexaenoic acid, a supplement that phosphorylates nNOS (i.e., inhibits nNOS activity) protects dopaminergic neurons from MPTP toxicity in rodent models of PD^15^. In addition, while a number of nNOS interacting proteins have been identified, HSP90 (heat-shock protein 90) has been demonstrated to be an important regulator of protein homeostasis and plays a specific role in preventing aSyn aggregation^16^ ascribing it significance in PD. While these findings seem to suggest that HSP90 may have a protective role in PD, HSP90 has also been shown to interact with and amplify nNOS activity^17^ thereby contributing to RNS. In cell models of PD, HSP90 inhibitors have been shown to protect against PD-related phenotype induction by preventing neurite loss and even aSyn aggregation^18,19^. Likewise, knockdown of nNOS using siRNAs protects dopamine neurons in cell and animal models of PD^20^. Together these findings suggest that nNOS is upregulated in PD and that suppression of nNOS activity may prevent PD pathogenesis.

Another relevant isoform of NOS is inducible nitric oxide synthase (iNOS), which is expressed by glial cells such as astrocytes and macrophages. iNOS is an enzyme ~131 kDa in size, encoded by the NOS2 gene. Like nNOS, iNOS consists of two domains; an oxygenase and reductase domain which facilitates the binding of calmodulin and the subsequent production of NO through a series of electron transport events. Its activity is regulated by protein interactions (e.g., kalirin), substrate and co-factor availability (cationic amino acid transporter L-arginine). Unlike the constitutive expression of nNOS in neurons, iNOS is stimulated, hence the term “inducible”. An operative distinction between iNOS and nNOS is the ability of iNOS to bind to calmodulin at much lower concentrations of calcium making NO production from iNOS tenfold that of nNOS-NO production^21^.

Elevated levels of iNOS have been found post-mortem in multiple neurodegenerative diseases including Parkinson’s-patient brains^22^. In support of this observation, there is heightened iNOS expression in multiple animal models of PD using 6-OHDA^23^, MPTP^24^, and even aSyn oligomers^25^ whereas mice lacking iNOS are resistant to many PD-inducing stressors^26^. Moreover, glial cells readily populate brain regions with active neurodegeneration, increasing the potential for high NO levels that can further exacerbate toxicity in surrounding cells.

### Nitrate reduction as a means of RNS generation

Independently of NOS family members, nitrate (NO_3_^−^) and nitrite (NO_2_^−^) can be reduced to NO or other RNS. Nitrates and nitrites are commonly sourced from diet, most notably vegetables. The reduction of NO_3_^−^ to NO_2_^−^ is most frequently catalyzed by the gastro-intestinal microbiome which uses NO_2_^−^ to produce energy and, in turn, generates nitrogen oxide by-products that can be absorbed into the body via the GI-tract. Alternatively, the reduction of NO_3_^−^ can be catalyzed by mammalian NO_3_^−^ reductases (e.g., Xanthine oxidase)^27^ or in mitochondria at complex III/ubiquinol complex^28^. NO_2_^−^, utilized as an NO reserve, is circulated throughout the body and further reduced to NO with the help of enzymes (*eg*. xanthine oxidoreductase, aldehyde oxidase), carrier proteins (e.g., deoxyhemoglobin, neuroglobin), or co-factors (e.g., ascorbic acid) in various tissues. Indeed, dietary NO_3_^−^ increases NO_2_^−^ availability to the brain^29^ where high levels of ascorbate might facilitate the conversion of NO_2_^−^ to NO^30^. It has been further hypothesized that NO_2_^−^ reduction in the brain may replace NOS-derived NO production when NOS is compromised, as in states of hypoxia or ischemia^31^. This event may occur at mitochondria^32^. NO may further react with other nitrogen oxide, oxide, or superoxide radicals to produce the potent nitrating agents ONOO^−^, N_2_O_3_, or NO_2_. Whether dietary-derived NO_3_^−^ or NO_2_^−^ contribute significantly to NO in the brain remains unclear, yet a link between gut microbiome and phenotypic PD-onset has recently been proposed^33,34^.

It is reported that PD patients have altered gut microbial ecosystems relative to healthy controls. More specifically, PD patients have decrease short chain fatty acids (causing decreased vitamin levels) as well as small intestinal bacterial overgrowth, which perpetuates oxidative and nitrosative stress in the gut. Patients also have increased levels of gut-aSyn relative to healthy controls, and this is observed years before motor symptoms arise^35^. Indeed, in the last decade several groups have put forth studies that support the theory that PD originates in the gut and that overtime pathology spreads into the brain. This is believed to be possible through the connectivity of the enteric neurons located in the gastrointestinal wall and the central nervous system. The theory postulates that external stressors stimulate an immune response in the gut, which triggers and seeds pathology from the enteric system to the brain via the vagal nerve^33^. In support of this, evidence suggests that pathological (or misfolded) aSyn inoculated into the duodenum can spread from the gut into the brain of rodents^36^ and that severing the vagal nerve inhibits this phenomenon^37^. Furthermore, oral administration of the pesticide rotenone triggers synucleinopathy, which spreads from the GI into the brain of inoculated mice^34^. This indicates that toxins that elicit an oxidative stress response in the gut are capable of triggering PD related synucleinopathy. A study by Sampson et al. suggested that gut microbiota may dictate PD-motor phenotypes; as PD-patient fecal transplants into germ free animals triggered locomotor deficits, whereas fecal transplants from healthy human donors had no effect on locomotor function. In the same study, Sampson et al. demonstrated that synucleinopathy itself was markedly reduced in germ-free mice despite aSyn overexpression^38^. As gut-brain connectivity is bi-directional, it is plausible that PD pathologies might also spread from the CNS into the gut. Indeed, 6-OHDA induced nigral-striatal brain lesions caused a reduction in fecal output^39^, further asserting a relationship between the gut and brain. Moreover, transgenic human-aSyn and MPTP-induced murine models of PD display GI-dysfunction such as constipation in parallel with aSyn accumulation^40^. Fecal transplantation to MPTP-induced PD mice reduced PD-phenotypes^41^ suggesting that unidirectional gut-to-brain communication predominates with respect to the influence on motor phenotypes. It is therefore interesting to speculate as to whether NO_3_^−^ or NO_2_^−^ imbalance in the gut contributes to the spread of synucleinopathy from the enteric nervous system to the central nervous system.

## RNS-induced pre-degenerative dysfunction of dopaminergic neurons

### NO-mediated alterations axo-dendritic function

**Fig. 3:**
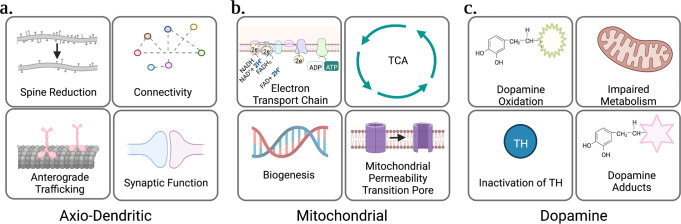
RNS-induced dysfunctions in Parkinson’s disease. This schematic highlights the major phenotypes that arise as a consequence of RNS in PD. This includes axo-dendritic defects (**a**), mitochondrial defects (**b**) and dopamine-induced defects (**c**) discussed in this review. This figure was created using Biorender.

As discussed above, nNOS contains a PDZ domain that confers binding capacity to many post-synaptic density-proteins (e.g., PSD93/95, PFK-M, CAPON, and syntrophin) and is therefore regionally distributed along synaptic spines. This localization is key to its canonical function, which is to promote NO-mediated second messenger signaling. In this way, NO reacts with guanylyl cyclase to produce cyclic guanosine monophosphate which is important for regulating neuronal dynamics including outgrowth and synaptic plasticity^42^. In addition, NO has a major role in regulating synaptic activity through s-nitrosylation and/or transnitrosylation of proteins^43–45^. In the healthy state, NO protects neurons from hyperexcitability by s-nitrosylating and inhibiting NMDARs (SNO-NMDAR)^46^. Choi et al. were the first to describe the mechanism whereby NO inhibits the NMDAR via s-nitrosylation of cysteine 399 on the NMDAR-subunit NR2A^46^. NO has also been shown to protect neurons from hyperexcitability by regulating AMPAR expression, a major component of ionotropic glutamate signaling. AMPAR surface expression is increased (SNO-Stargazin) or decreased (SNO-Thorase) in response to S-nitrosylation of respective regulatory proteins^47,48^. Moreover, in neurons NO itself can act as a neurotransmitter; regulating calcium signaling^49^, stimulating extracellular vesicle release and endocytosis^50^, and is even involved with learning and memory formation through its activation of CREB^51^ and retrograde transmission from the post- to pre-synaptic terminal, stimulating neurotransmitter release and long-term potentiation^52^. However, when in excess, as is the case in PD-neurons, NO alters axo-dendritic function impairing synaptic signaling, vesicular trafficking, and dopamine homeostasis (Fig. 3).

The aberrant s-nitrosylation of synaptic proteins is believed to lead to the dysregulation of synaptic activity in PD-neurons. While the exact effects of superfluous NO or its derivatives on synaptic function in PD have not been fully elucidated, there is evidence that excess NO alters axo-dendritic function, specifically impairing neurite length through the S-nitrosylation of protein disulfide isomerase or microtubule associated protein 1b, and causing synaptic spine loss through the hyperactivity of Cyclic-dependent kinase 5 (CDK5) following CDK5 S-nitrosylation^53,54^. These axo-dendritic impairments alter network connectivity, which is associated with cognitive decline and even neuronal death. NO also regulates NMDAR-excitotoxicity^55^ that can be further exacerbated by SNO-Src and SNO-SHP-2^56,57^. Src, a family of proteins tyrosine kinases, was found to be activated by autophosphorylation and S-nitrosylation leading to the phosphorylation of NMDAR subunit NR2B and the subsequent increase of NMDAR activity. Additionally, NMDAR excitotoxicity is also regulated by SHP-2, a Src homology-2 domain containing phosphatase. SHP-2 has been shown to promote cell survival via ERK1/2 pathway, while nitrosylation of SHP-2 inhibits its phosphatase activity. Indeed, NMDAR-dependent calcium influx triggers nNOS to generate NO thereby compounding the effects of SNO-Src and SNO-SHP-2 and further contributing to NMDAR-mediated excitotoxicity. Moreover, NO has been shown to induce the synthesis of NO per se^58^, indicating that the superfluous levels of NO in PD might lead to a vicious loop of NO production.

Recent evidence suggests another important function of NO is to regulate intracellular trafficking of vesicles and cargo. We and others have shown that PD patient derived dopaminergic neurons are particularly susceptible to nitrosative stress^59,60^. These studies demonstrated that low doses of agrochemicals, which had no effect on isogenic control neurons, caused the nitration of tubulin and impaired KIF5b transport of mitochondria in neurons harboring aSyn mutations^61^. Moreover, Redondo et al. show that NO reduces KIF5A and KIF21B expression in neurons^62^, suggesting that an upregulation of NO in PD would cause a reduction of anterograde trafficking. The loss of kinesin-driven axonal transport would likely have many consequences for neurons such as (1) energetic deficits given that cells would have impairments in transport of metabolic enzymes. This defect would particularly affect energy production at the synapse where there exists a large energetic demand. We can speculate that another consequence of decreased transport in neurons would be (2) reduced neurotransmitter production, given that enzymes that synthesize neurotransmitters are required to be trafficked to the synapse via vesicles. The latter defect may be exacerbated by the S-nitrosylation of VMAT2 (vesicular monoamine transporter)^63^, which has been shown to be important for the transport of enzymes responsible for dopamine synthesis (e.g., tyrosine hydroxylase and AADC) as well as important for storing dopamine in vesicles to protect dopamine from oxidation and degradation. Ultimately, Redondo et al. show that the loss of kinesin proteins led to a reduction in axonal length and neuron survival^62^.

### Effects of NO on mitochondrial function in neurons

In a healthy system NO has been ascribed a role in regulating mitochondrial function. In 1994, Brown and Cooper provided evidence that NO alters mitochondrial respiration. They showed that in isolated synaptosomes low levels of NO compete with oxygen to inhibit or activate cytochrome c, respectively^64^. Since mitochondria cluster to regions where the demand for ATP is heightened, such as the synapse, it is possible that NO regulates mitochondrial oxygen consumption in order to maintain ATP supply. This regulation would be particularly important in the context of substantia-nigral dopaminergic neurons given their complex arborization and pace-making activity. Moreover, substantia-nigral dopaminergic neurons have fast spiking Ca^2+^ transients that increase the intracellular concentration of Ca^2+^ that is subsequently buffered, in part, by mitochondria. Not only does this put mitochondria at risk for Ca^2+^ overload leading to excitotoxicity and/or apoptosis, but Ca^2+^ that is not buffered may further stimulate nNOS-generated NO. Together, mitochondrial- NO reacts with superoxide produced by mitochondria to promote the production of the highly toxic ONOO^−^. Studies have shown that ONOO^−^ can impair mitochondrial-ATP synthesis through inhibition of mitochondrial respiratory complexes, mitochondrial polarization, and detoxifying enzymes in addition to promoting mitophagy^65,66^.

Many mitochondrial-related proteins are reported to be S-nitrosylated, both endogenously and in response to stimuli. Indeed, Chang et al. used the biotin-switch LC-MS/MS method to detect S-nitrosylated proteins in isolated rat brain-derived mitochondria identifying s-nitrosylated mitochondrial targets that include: pyruvate dehydrogenase, ﻿succinyl-CoA ligase, complex I, VDAC (voltage dependent anion channel), and prohibitin (PHB)^67^. Still others have performed various SNO-capture methods (SNOSID, SNOTrap, Phenylmercury (MRC)) on whole brain extracts or primary cultured neurons to generate global lists of SNO-modified proteins. While the implications of each of these modifications have not been fully elucidated, the exuberant increase in RNS in PD would likely increase the number of mitochondrial targets nitrosylated in the disease setting. Studies indicate that the S-nitrosylation of the electron transport chain, the citric acid cycle, carnitine/acylcarnitine transporter and the nitration of HSP90 impairs metabolism^68,69^. Together, these studies suggest that RNS-altered mitochondrial targets leads to a loss of function. On the other hand, the nitrosylation of voltage-dependent anion channel 1, a component of the mitochondrial permeability transition pore, is believed to trigger the opening of the channel leading to a gain of function^67^. Consequently, this leads to Ca_2_^+^ dyshomeostasis and mitochondrial depolarization, cytochrome *c* release and apopotosome formation, permeabilization of the outer mitochondrial membrane as well as causing the hyperproduction and release of O_2_^−^ and H_2_O_2_ leading to other free radical damage^70^. It has, however, been reported that the effects of NO on VDAC is concentration dependent: low doses of NO block the channel, while high doses of NO open the channel^71^. While we have just described some of the deleterious effect of RNS on mitochondrial proteins, the specific effects in PD remain poorly characterized.

In addition to mitochondrial proteins that can be nitrosylated, extra-mitochondrial proteins that affect mitochondrial function are susceptible to nitrosylation or nitration leading to altered mitochondrial dynamics. For instance, while nitrosylation of CREB may enhance its ability to bind DNA and leads to the upregulation of mitochondrial-biogenesis genes^72^, the nitration of PPAR has been shown to impede the expression of mitochondrial proteins^73^. Parkin (i.e., Park2) is an E3 ubiquitin ligase that is believed to play an important role in directing misfolded proteins for degradation via the ubiquitin-protease system^74^ and helps regulate mitochondrial dynamics through mitochondrial biogenesis, import of mitochondrial proteins and mitophagy (targeting mitochondria to phagosomes)^75^. In humans, Parkin mutations are associated with autosomal recessive familial PD. Moreover, SNO-Parkin is more present in PD patients and in rotenone or MPTP-treated mice than controls^76,77^. SNO-Parkin leads to mitochondrial depolarization whereas SNO-PINK1 decreases Parkin translocation to mitochondrial membranes, disrupting mitophagy^78,79^. In addition, the S-nitrosylation of the mitochondrial chaperone protein PHB has actually been shown to be neuroprotective against stress evoked by oxygen and glucose deprivation^80^; however, PHB’s expression is reportedly reduced in PD patient brains^81^ suggesting PD neurons are more susceptible to this stress. In PD patient brain tissue and cellular models of PD, S-nitrosylation of peroxiredoxin (PrxII) decreases peroxidase activity causing H_2_O_2_ to accumulate, exasperating oxidative stress^82^.

### Effects of NO on dopamine homeostasis in neurons

The unique susceptibility of dopaminergic neurons to nitrosative stress, may relate to dopamine synthesis and catabolism. Indeed, the dopamine synthesizing enzyme tyrosine hydroxylase can be inactivated by tyrosine nitration, reducing intracellular dopamine levels^83,84^. In addition, NO also catalyzes the auto-oxidation of dopamine (via quinones and semiquinones)^85^ causing protein-, lipid-, or DNA- dopamine-adducts that can lead to neurodegeneration. What’s more, dopamine itself can react with many RNS including nitric dioxide, nitrogen dioxide and dinitrogen trioxide forming 6-nitrosodopamine which can further react with RNS to produce 6-nitrodopamine which can then produce other radicals and RNS^86^. Together these findings suggest that dopaminergic neurons are vulnerable to nitrosative stress due, in part, to the presence of dopamine per se. RNS-altered dopamine may dysregulate dopamine homeostasis and further intensify RNS.

The effects of RNS on mitochondria also has specific implications for dopaminergic neurons since the breakdown of dopamine by the mitochondrial localized enzyme monoamine oxidase produces the metabolite 3,4-dihydroxyphenylacetic acid (DOPAC) whose interaction with NO can impair mitochondrial respiration^87^. The combination of DOPAC and NO was shown to also cause a decrease in glutathione^88^. Glutathione, a thiol-reducing agent and antioxidant responsible for the breakdown of H_2_O_2_, is known to be dysregulated in PD patients. Indeed, decreasing glutathione synthesis in dopamine cells causes the inhibition of mitochondrial complex I in either a NO- or ONOO^−^-dependent manner, depending on whether the treatment was acute or chronic, respectively^89,90^. While the nature of the interaction between NO, DOPAC and complex I remains elusive, it is likely to play a part in the early decline of dopaminergic neurons in PD.

## Role of RNS in protein misfolding and Lewy Body deposition

### Alpha-Synuclein protofibril formation in PD

Many causal aSyn mutations have been identified (A30P, A53T, E46K, G51D, duplication, and triplication) in PD patients. While familial forms of PD only account for about 10% of cases, the presence of aSyn in Lewy-bodies of sporadic PD cases highlights its importance to multiple disease stratifications. Although the processes driving Lewy body formation are not well understood, much is known about how aSyn fibrillization contributes to Lewy Body pathology. aSyn is a 14 kDa protein consisting of three domains: the amino-terminus, the non-amyloid β-component (NAC), and the carboxyl-terminal domain. In solution, aSyn exists primarily as an unstructured random-coil. Since the amino-terminus is amphipathic, consisting of seven KTKEGV repeat sequences, aSyn reconfigures into an α-helical form when bound to anionic phospholipid membranes. Although the function of aSyn remains poorly characterized, most evidence supports functions at the synapse, regulating vesicle storage, dopamine synthesis and neurotransmission. When misfolded, aSyn represents the major component of β-sheet-rich cytoplasmic inclusions within Lewy bodies, which accumulate prior to neurodegeneration in PD. Prior to deposition into Lewy bodies, aSyn protofibrils (or oligomers) accumulate. In PD, aSyn oligomers are conformationally self-templating and able to cross-seed with other structural conformers of aSyn^91^. What’s more, oligomers actively recruit other proteins into aggregates^92^. aSyn oligomers can also propagate between cells through synaptically linked regions of the brain^93,94^, exo-endocytosis^95^, tunneling nanotubes^96^ or by receptor mediation (e.g., Lag3)^97^ leading to the pathological spread of synucleinopathy. aSyn oligomers can also permeabilize lipid membranes^98^, trigger mitochondrial dysfunction^99^, proteostatic stress^100–102^, and impair synaptic function^103^. Although the pathological progression of aSyn and Lewy Body pathology can be variable, the patterning is typically predictive: beginning in the medulla, continuing into the midbrain, and finally infecting the cortex^104^. It remains controversial whether aSyn per-se or mature Lewy bodies ultimately trigger cell death. While Lewy-bodies appear to trigger cell death by blocking intracellular-transport as well as impairing mitochondrial and synaptic function, they may develop in response to sequestration of aSyn oligomers that are highly toxic.

### RNS potentiates aSyn misfolding and pathological deposition

Nitration of aSyn can potentiate aSyn-oligomer formation. Oxidative modification to tyrosine can occur in one of two ways: a hydrogen atom in the 3′ position of a tyrosine ring can be replaced with a nitro-group (3-Nitro-Tyrosine (3NT)), or tyrosines can react with each other to form 3,3′-dityrosine crosslinks. Exposure of aSyn to nitrating agents can encourage o,o′-dityrosine crosslinking between the N-domain tyrosine (Y39) and C-terminal tyrosines (Y125, Y133, Y136) to generate aSyn-dimers that are more prone to oligomer formation^105–108^. Perhaps unsurprisingly, mutated aSyn (A30P and A53T) has an increased propensity for dityrosine cross-linking^107^. Additionally, tyrosines play an important role in aSyn-vesicle binding while nitration impairs this interaction (making the charge more negative at the N-terminal domain, or causing a conformational change when the C-terminal domain is nitrated) thereby increasing the amount of aSyn in a random coil or beta-sheet conformation and thus shifting equilibrium toward oligomer formation^105,106,109^. It has also been suggested that nitrated aSyn promotes the seeding of aSyn-oligomers from cell-to-cell^110^.

There exists evidence that nitration of aSyn can also potentiate fibril formation. In cells exposed to ONOO^−^, formation of aSyn aggregates is dependent on nitro-tyrosine adduct formation^111^, supporting the notion that that protein nitration may serve as a biomarker of PD. Indeed, nitrated aSyn is almost exclusively found in the insoluble protein fraction making aSyn more resistant to degradation, more compact, and more stable^112,113^. Moreover, mutating aSyn-tyrosine Y39 residue to phenylalanine reduces the ability of aSyn to fibrilize^105,113^. Many studies have also demonstrated that nitrating-aSyn through the addition of nitrating agents (eg. ONOO^−^ or TMN) promotes non-amyloidogenic β-sheet oligomers and dimers, as opposed to fibrils^105,112–114^. In this way, it has been suggested that nitration of aSyn might occur post-fibril-formation and function to stabilize aSyn in its new form. Taken together, these studies suggest that tyrosine nitration can augment fibril formation by promoting dityrosine crosslinking capable of seeding pathology and may in addition represent a post-fibril modification that functions to stabilize fibrils.

In PD patients, aSyn itself was shown to be at least one target of nitration in PD-patient brain samples. Indeed, higher RNS and nitrated aSyn in PD-patient serum levels correlates with worsened PD-related outcomes^115^. Moreover, nitrated aSyn was specifically identified within Lewy-body-like and insoluble inclusions^116^. Overexpression of aSyn in HEK293 cells lead to intracellular inclusions following exposure to a NO donor and oxidizing agents or ONOO^−^^111,117^. Likewise, in mice treated with MPTP, which causes the degeneration of dopaminergic neurons, immunoprecipitation of aSyn and subsequent western-blot analysis indicated that aSyn was nitrated as early as 4 h following MPTP treatment^117^. Despite the fact that very few proteins undergo nitration, increased levels of nitrated-aSyn in PD patients suggests that nitration might be linked to PD-pathology.

Nitrated-aSyn likely has many functional consequences. An in vitro assay by Mishizen-Eberz et al. demonstrated that nitrated aSyn fibrils were able to be cleaved by calpain1 just as un-nitrated aSyn-fibrils were. However, the authors noted that the cleaved fibrils from nitrated-aSyn formed uniquely structured fragments which were wider and had a more exposed NAC domain relative to the cleavage of un-nitrated aSyn fibrils. In addition, the nitrated-aSyn fragments promoted the recruitment of soluble aSyn into its aggregate^118^, suggesting that the nitration of aSyn might seed pathology more efficiently than non-nitrated aSyn fibril-fragments. Moreover, nitration of aSyn has been shown to increase the rate of fibrillization and simultaneously slow the rate of proteolytic degradation (e.g., cleavage by calpain1)^106^. In addition, when cells and mice were exposed to aSyn or nitrated-aSyn, the nitrated aSyn was more toxic as it causes dopamine-cells to degenerate and mice to perform more poorly on behavioral motor-coordination assays^119^.

### Increased aSyn correlates with increases in RNS

There exists evidence that strongly suggests a positive-feedback correlation between aSyn and RNS. Several reports have determined that aSyn accumulation leads to increased NO generation and s-nitrosylation of proteins implicated in PD pathogenesis. In brains of sporadic PD patients with diffuse Lewy bodies, s-nitrosylation of Parkin^120^, p53^120^, PTEN, and DJ-1^121^ have been reported that result from an interlinked mechanism of trans-nitrosylation leading to degeneration of nigral neurons. aSyn overexpressing transgenic mice show elevated levels of S-nitrosylated Parkin and PINK1 and defects in mitochondrial quality control^79^, whereas aSyn-knockout cells and mice are resistant to the deleterious effects of RNS elicited by exposure to MPP+ or LPS^122^. That parkin is heavily nitrosylated in models of aSyn overexpression is perhaps not surprising given reports that Parkin cysteine oxidation is linked to redox balance in human midbrain^123^ and that Parkin suppresses unfolded protein stress-induced cell death^124^. Studies from human stem-cell-derived dopaminergic neurons have shown that neurons harboring the endogenous aSyn-A53T mutation have increased kinetics of NO synthesis and are more susceptible to RNS-toxins^59,61^. Moreover, mutant aSyn (A53T and E46K) neurons have increased basal levels of S-nitrosylated proteins relative to isogenic controls. In fact, when considering reports on the constituents of Lewy Bodies^125^, one third of the proteins deposited are modified by S-nitrosylation (Table 1). With respect to protein nitration, Tapias et al. showed that inoculation of aSyn preformed fibrils into mice and nonhuman primate brains caused an accumulation of 3NT-modified proteins, particularly in dopaminergic neurons^25^. Together, these findings suggest crosstalk between aSyn and RNS with respect to pathological deposition of protein aggregates in PD.

**Table 1:** Identified constituents of Lewy Bodies that are prone to S-nitrosylation.

Protein Name	Evidence of SNO modification	UniProt - Function
14-3-3 protein epsilon	^161,162^	Adapter protein
Actin, cytoplasmic 1	^163–167^	Produces filamentous networks, aids in cell motility
Alpha-internexin	^168–171^	Intermediate filament, important for neuronal cytoskeleton structure
ATP synthase beta chain, mitochondrial precursor	^165,172^	Part of the mitochondrial-electron transport chain that generates ATP
Beta tubulin	^161,170,173,174^	Major component of microtubules
Clathrin heavy Chain 1	^161,170^	A major constituent of the polyhedral coating on vesicles, plays a role in autophagosome formation
Creatine kinase, B chain	^161,163,166,170,172^	Transfers phosphate between ATP and phosphogens
DNM1 protein	^175^	Mediates mitochondrial membrane fission
Dynamin 3	^175^	Aids in microtubule bundling and likely vesicular trafficking
Dynein, cytoplasmic, heavy polypeptide 1	^170^	Retrograde transport
Glyceraldehyde-3-phosphate dehydrogenase	^161,163,165,176–178^	Plays a role in glycolysis, modulates cytoskeleton assembly, signals nuclear target proteins
Gelsolin precursor	^165^	Regulates actin assembly
Glucose phosphate isomerase	^179^	Part of the glycolysis pathway
Heat shock protein 86 (HSP90)	^170,180^	Molecular chaperone and transcriptional modulator
Isocitrate dehydrogenase [NAD] subunit alpha, mitochondrial precursor	^170,181^	Subunit of the enzyme that catalyzes the decarboxylation of isocitrate into alpha-ketoglutarate
Microtubule-associated protein 1B	^54,170,176,182^	Helps regulate microtubule dynamics
Microtubule-associated protein tau isoform 2	^176,183^	Helps in microtubule assembly and stability
Neurofilament light polypeptide	^167^	Intermediate neurofilament, a biomarker of axonal damage
Neurofilament 3 (150 kDa medium)	^166,167^	Structural component of the cytoskeleton
Plectin 6	^170,184^	Interlinks intermediate filaments with microtubules and microfilaments and anchors intermediate filaments to desmosomes or hemidesmosomes.
Sodium/potassium-transporting ATPase alpha-2 chain precursor	^161,163,167,176^	Catalytic component of the ATPase.
Spectrin alpha chain, brain	^167,170,185^	Calcium-dependent movement of the cytoskeleton. Molecular scaffold protein. Links the plasma membrane to the cytoskeleton.
Splice Isoform 1 of Clathrin heavy chain 2	^170^	A major constituent of the polyhedral coating on vesicles, plays a role in autophagosome formation
Splice Isoform 1 of Desmoplakin	^165^	Links intermediate filaments, actin and microtubule networks.
Splice Isoform 1 of Dynamin 2	^175,186^	Ubiquitously expressed. Aids in microtubule bundling and likely vesicular trafficking
Splice Isoform 1 of Heat shock cognate 71 kDa protein (HSC70)	^161–163,167,170^	Molecular chaperone that works with co-chaperones to activate proteolysis, refolds and transports proteins and even targets proteins for degradation.
Splice Isoform 1 of Microtubule-associated protein 2	^176^	Stabilizes microtubules.
Splice Isoform 1 of Plectin 1	^170,184^	Interlinks intermediate filaments with microtubules and microfilaments and anchors intermediate filaments to desmosomes or hemidesmosomes.
Splice Isoform 1 of Spectrin beta chain, brain 1	^170,185^	Calcium-dependent movement of the cytoskeleton. Molecular scaffold protein. Links the plasma membrane to the cytoskeleton.
Splice Isoform 1 of Voltage-dependent anion-selective channel protein 2	^165,170,176^	Mitochondrial outer membrane channel. Alters conformation (open/closed) in response to the mitochondrial membrane potential.
Splice Isoform 2 of Microtubule-associated protein tau	^183^	Promotes microtubule assembly and stability.
Synaptotagmin-1	^161,163^	Calcium sensor that triggers neurotransmitter release at the synapse. May play a role in synaptic vesicle trafficking.
Tubulin alpha-1 chain	^161,167,173,174,176,187^	Major constituent of microtubules.
Tubulin beta-2 chain	^161,166,167,173,174,176^	Major constituent of microtubules.
Ubiquitin-activating enzyme E1	^170,188^	Activates ubiquitin and conjugates it to targeted proteins during ubiquitinylation.
Ubiquitin carboxyl-terminal hydrolase isozyme L1	^161,176^	A deubiquitinating enzyme.
Vimentin	^170,180,189–191^	An intermediate filament that acts as a scaffold protein for the nucleus, endoplasmic reticulum and mitochondria and the cytoskeleton.

## Targeting RNS as a PD therapy

### Reducing RNS by targeting NOS

Upregulating proteins that interact with or inhibit nNOS activity may represent a means of mitigating PD pathogenesis. The specific nNOS inhibitor 7-nitroindazole protects against dopaminergic neuron depletion in animals treated with 6OHDA^126^ or MPTP^12^, even preventing motor impairments such as apomorphine induced contralateral rotations^127^ and catalepsy^12^. nNOS inhibition by N_w_nitro-L-arginine methyl ester can reduce NO accumulation in human induced pluripotent stem cell (hiPSC)-derived PD neurons (harboring the A53T-SNCA mutation) protecting neurons from mitochondrial dysfunction^61^ and rescuing axodendritic pathology^128^. Likewise, treatment with docosahexaenoic acid causes the phosphorylation of nNOS that in turn decreases nNOS activity preventing PD-like motor deficits in MPTP treatment in mice^15^. While inhibiting NOS may seem to be an obvious therapy, this is complicated by the fact that nitric oxide is integral for physiological function, particularly for the vascular system. In addition, NO inhibition has been shown to be related to insulin resistance and can cause other adverse side effects.

As an alternative to NOS inhibition, gene silencing by interfering RNAs (iRNAs) could degrade RNAs that are integral for NOS synthesis. iRNAs are delivered to cells through viral (e.g., nanoparticle or liposomes) non-viral vectors (e.g., adeno-associated virus or retrovirus). Similar to the protection from PD-phenotypes exhibited by nNOS knockout mice^129^, striatal inoculation of iRNA targeting-nNOS reduced 6-OHDA toxicity by preventing dopaminergic neuron degeneration and behavioral impairments in rats^20^. Clinical trials are ongoing that employ the use of iRNAs for PD, thus specific targeting of NOS in the SNpc to decrease RNS may be feasible in the future.

### Antioxidants to reduce RNS

Some studies have shown that scavenging RNS may also be one way to attenuate PD pathologies. One way to accomplish this is to inhibit NADPH oxidase (NOX), which is responsible for the formation of O_2_^−^ and, subsequently, the formation of ONOO^−^. In vitro experiments by Schildknecht et al. showed that the NOX inhibitor GTK136901 reduced the amount of ONOO^−^, prevented alpha-synuclein nitration, and protected human dopaminergic cells from ONOO^−^ toxicity^130^. Similarly, minocyclin, a scavenger of ONOO^−^ [194], has also been shown to reduce ONOO^−^ in a LPS model of PD, preventing 3-NT immunoreactivity^131^. Moreover, minocyclin has also been shown to prevent 6-OHDA and MPTP dopaminergic neuronal degeneration though its inhibition of iNOS-produced NO^132,133^. Together, these findings suggest a dual-role mechanism of action for minocyclin (1) scavenging ONOO^−^ and (2) inhibiting iNOS in glial cells. Indeed, a number of other NOX inhibitors are being studied for their therapeutic potential in PD^134^.

**Fig. 4:**
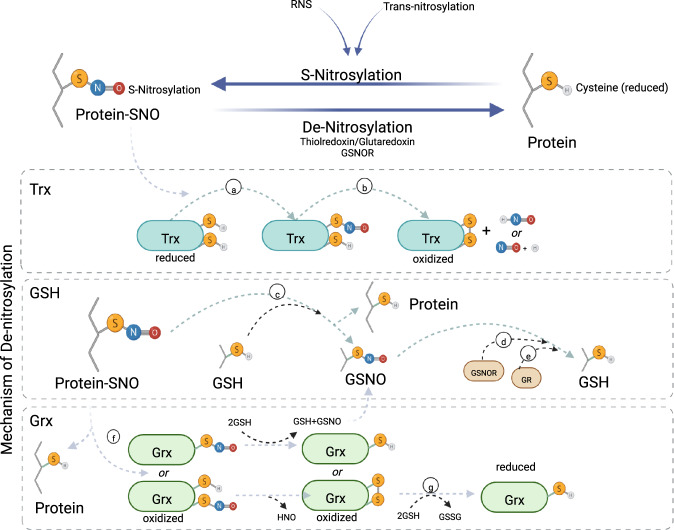
Major pathways of protein de-nitrosylation. Two major de-nitrosylation pathways reduce s-nitrosocysteine in cells: the thioredoxin reductase (Trx) family of redox sensors that includes Glutaredoxin (Grx), and the indirect S-nitrosoglutathione reductase (GSNOR) system. **a** Trx removes the s-nitrosyl-group from the protein-SNO (either through Trx-mediated trans-nitrosylation or Trx-disulfide-bond formation within the protein-SNO) and then **b** generates a disulfide bond, freeing HON or NO + H^+^. Alternatively, the GSNOR system **c** first requires that glutathione (GSH) react with the s-nitrosocysteine of an S-nitrosylated protein, transferring the nitrosyl-group to the thiol of the cysteinyl-group of GSH generating GSNO. **d** Next, the enzyme S-nitrosoglutathione reductase (GSNOR) and co-enzyme NADH catalyzes the conversion of GSNO to an N-hydrosulfinamide (GSNOH) intermediate and subsequently to glutathione-disulfide (protein-GSSG) and hydroxylamine (not shown). **e** Protein-GSSG is then reduced to GSH through the activity of glutathione reductase (GR) and co-enzyme NADPH. **f** The Grx member of the Trx family has two modes of SNO reduction. Grx can form an oxidized Grx intermediate (Grx-SNO) on one cysteine residue following transfer of the nitrosyl-group from protein-SNO. This can either be reduced though the GSH pathway described above (mono-thiol reaction) or through the traditional Trx system (diol-thiol reaction) by producing a disulfide bond. **g** The now oxidized Grx is reduced by two GSH molecules to restore the cycle. This figure was created using Biorender.

There has been some interest in repurposing the drugs simvastatin (i.e., Zocor) or dimethyl fumarate (i.e., Tecfidera) for PD. These drugs are relevant as they function to activate NRF2 which upregulates antioxidant response enzymes to mitigate ROS/RNS^135,136^. NRF2 is released from its repressor (KEAP1) allowing NRF2 to translocate to the nucleus and transcribe >800 genes, including phase i enzymes involved in the metabolic detoxification pathway and phase ii conjugation enzymes that metabolize functional groups. Particularly important to the regulation of RNS, NRF2 activity upregulates enzymes such as thioredoxin and GCLC, the rate limiting enzyme in glutathione synthesis. Glutathione can react with NO to form GSNO which acts as an NO reserve. Glutathione also regulates GSNO(R) reductase (i.e., AHD5) which, together with thioredoxin, reduces protein S-nitrosothiol groups (Fig. 4). Studies suggest that both simvastatin and dimethyl fumarate are neuroprotective in animal models of PD^137–141^. Indeed, dimethyl fumarate can rescue antioxidant enzyme expression as well as increase neuritic arborization and complexity in hiPSC-derived dopamine neurons harboring the A53T aSyn mutation^128^. While results from the Simvastatin clinical trial have yet to be reported and trials using dimethyl fumarate have yet to commence, there is reason to be hopeful that a drug targeting RNS may be effective for PD therapy.

Additional clinical trials have aimed to improve the antioxidant response in PD, including the study of lithium, hydrogen, Deferiprone and N-acetylcysteine. The aforementioned drugs exhibit neuroprotective actions in PD models; reducing oxidative/nitrosative stress, aSyn aggregation or nitration, and activating anti-apoptotic pathways^142–150^. While early findings from clinical trials show that the protective effects of hydrogen and deferiprone did not reach statistical significance^151–153^, N-acetylcysteine (NAC) shows promising efficacy. NAC is a membrane-permeable form of cysteine, a precursor for glutathione. In vitro studies using PFFs to induce synucleinopathy in primary neurons showed that treatment with NAC reduced aSyn aggregates and oxidative stress^154^. Likewise, many animal models of PD show that oral administration of NAC rescued dopaminergic neuron loss, reduced oxidative stress, and improved motor outcomes^154–157^. To date, clinical trials investigating the effectiveness of NAC demonstrate that the oral coupled with intravenous administration of NAC increases the expression of the dopamine transporter and shows modest improvement in UPDRS scores in PD patients^158,159^. While these results suggest NAC-treatment may be one potential strategy for reducing RNS in PD, the limited bioavailability of oral NAC in brain tissue and need for intravenous administration may limit its widespread use.

### Targeting alternative de-nitrosylating proteins

Since the nitrosylation of proteins has deleterious effects with immediate implications for PD, targeting this modification may offer therapeutic benefit. For example, S-nitrosylation of peroxireductase (Prx2) has been shown to impair the H_2_O_2_ reductase capacity of Prx2, resulting in increased oxidative stress. SNO-Prx2 has been found to be more abundant in PD models than controls^82^. In light of this, preventing this and other SNO-modifications might be an important step in mitigating PD pathology. Sunico et al. identified that the overexpression of sulfiredoxin (Srxn1), an enzyme responsible for the de-nitrosylation of SNO-Prx2, protected dopaminergic neurons from the effects of paraquat/maneb treatment in both mice and hiPSC-derived dopaminergic neurons^120^.

Perhaps the most well-studied protein for its nitrosylated/de-nitrosylated function in PD is Parkin. The oxidation of Parkin leads to its aggregation which reduces its ability to act as a redox sponge^123^. Recent reports indicate that the protein DJ-1 regulates SNO-parkin (and the S-nitrosylation of other proteins) via trans-nitrosylation^78,121^ as recently reviewed by Sircar and colleagues^160^. Moreover, inactivating DJ-1 mimics PD-related mitochondrial dysfunction^78^. Besides regulating SNO-Parkin, SNO-Dj-1 itself also represents a potential PD therapeutic target since DJ-1 plays a role in activating the antioxidant response, cellular metabolism, cell survival, and redox homeostasis^160^. However, the specific function of SNO-Dj-1 vs. de-nitrosylated Dj-1 have yet to be elucidated. Future research that identifies supplementary de-nitrosylating proteins may provide valuable information for the removal of SNO-modification and rescuing PD pathology.

In summary, the effects of RNS in PD are multifaceted. Herein, we describe the sources of RNS in neurons and provide evidence that RNS is implicated in PD. Accumulating evidence suggest that RNS exacerbates the rate of disease progression by promoting aSyn misfolding and accelerating deposition of aSyn aggregates. The sheer number of s-nitrosylated proteins that deposit in Lewy Body aggregates suggests that RNS, once triggered, is accelerating not only aSyn aggregation but the aggregation of many proteins critical to neuronal survival and function. This culminates in (1) axo-dendritic pathology coupled to loss of synaptic function, (2) impaired mitochondrial dynamics coupled with altered energy homeostasis, and (3) impaired dopamine metabolism, all of which contribute to further RNS imbalance. Thus, reducing or controlling RNS accumulation early in disease etiology may have multimodal benefits to people with PD. This is supported by positive outcomes in clinical trials for NAC showing rescue of dopamine levels and improved function in patient cohorts. While NAC use is limited by its poor bioavailability in brain tissue, these results may indicate that de-nitrosylating proteins of the Trx family such as (Grx, Srxn1and Prx2) may represent a new class of disease modifying targets against PD. Future studies should explore whether de-nitrosylating proteins are targetable and effective in diseased populations.

## Data Availability

Data sharing is not applicable to this article as no datasets were generated or analyzed during the current study.

## Code availability

Code sharing is not applicable to this article as no datasets were generated or analyzed during the current study.
