# Author Correction: Nitrosative stress in Parkinson’s disease

**DOI:** 10.1038/s41531-022-00390-z

**Published:** 2022-09-19

**Authors:** Morgan G. Stykel, Scott D. Ryan

**Affiliations:** 1 The Department of Molecular and Cellular Biology, The University of Guelph, Guelph, ON N1G 2W1 ON Canada; 2 Neurodegenerative Disease Center, Scintillon Institute, 6868 Nancy Ridge Drive, San Diego, CA 92121 USA

**Keywords:** Parkinson's disease, Cellular neuroscience

Correction to: *npj Parkinson’s Disease* 10.1038/s41531-022-00370-3, published online 11 August 2022

In this article the two spelling errors in Fig. 4 labels were corrected “oxidzed to oxidized and Thx to Trx. The original article has been corrected.

